# The Mean Individual Biomass (MIB) of Ground Beetles (Carabidae): A Review of Its Application to Ecosystem Succession, Biodiversity, and Climate Change Research

**DOI:** 10.3390/insects16121191

**Published:** 2025-11-23

**Authors:** Katarzyna Szyszko-Podgórska

**Affiliations:** Department of Environmental Research, the Institute of Environmental Protection-National Research Institute, Słowicza 32, 02-170 Warsaw, Poland; katarzyna.podgorska@ios.edu.pl

**Keywords:** Carabidae, mean individual biomass (MIB), indicator of ecosystem responses, habitat quality, functional traits, length–mass models, early-warning signals

## Abstract

Bioindication is a key tool for monitoring habitat quality and ecosystem dynamics under increasing anthropogenic pressure. Among model organisms, ground beetles (Coleoptera: Carabidae) play a particularly important role, and one of the widely applied functional indicators describing their assemblage structure is the Mean Individual Biomass (MIB). Introduced in the 1980s, this index reflects the average body mass of Carabidae and allows assessment of successional stages. Its computational simplicity and intuitive interpretation have led to its application in forests, agricultural landscapes, post-industrial areas, and glacier forelands. This paper synthesizes the development and applications of the MIB, highlighting both its advantages and methodological limitations (including variability of length–mass models, seasonal activity patterns, and dependence on sampling methods). Particular attention is given to the potential of the MIB in the context of global environmental change, including its role as an indicator of ecosystem responses to climate change and processes related to soil carbon sequestration. Based on a literature review, future research directions are identified, encompassing methodological standardization, integration of MIB with other ecological and molecular indicators, and expansion of analyses to regions beyond Europe. By linking classical bioindication with ecosystem functioning studies, the MIB may serve as a universal tool for environmental monitoring and the assessment of ecosystem services under accelerated global change.

## Simple Summary

Monitoring changes in insect communities helps us understand how ecosystems respond to human activity and climate change. This review explains how a simple index, the Mean Individual Biomass (MIB), can be used to assess habitat quality based on the body mass of ground beetles (Carabidae). The method is easy to apply and provides an overview of environmental stability and disturbance. I summarize studies from forests, agricultural fields, post-industrial sites and glacier forelands showing that MIB increases in stable habitats and decreases when disturbance or habitat loss occurs. Although it cannot directly measure soil carbon storage, MIB reflects ecological processes that are important for biodiversity and ecosystem recovery. This knowledge can help improve environmental monitoring and restoration programs in Europe and beyond.

## 1. Introduction

Ground beetles (Coleoptera: Carabidae) are among the best-studied insect families in ecology and nature conservation. They comprise about 40,000 described species worldwide. According to global catalogs [1], more than 35,000 species have been listed, and currently about 38,600 valid names are recognized, with approximately 100 new species described each year [2]. Carabidae inhabit almost all terrestrial ecosystems, and their well-established taxonomy, ease of standardized sampling [3], and high sensitivity to environmental changes have made them a model group for studies on habitat quality, land use, and biodiversity [4].

The role of Carabidae as bioindicators has been widely confirmed. Numerous studies have demonstrated their sensitivity to disturbances in boreal and temperate forests, changes in agroecosystems, urbanization pressure, and successional processes in degraded areas [5,6,7,8,9]. Due to their close relationships with vegetation structure, soil conditions, and microclimate, ground beetles provide information not only about species diversity but also about ecological functions such as predation and participation in organic matter cycling [2,3,6].

Most studies on ground beetles (Carabidae) have focused on species composition, the identification of indicator species, or patterns of species diversity in forests [10,11,12,13,14], agricultural landscapes [15], and other habitats, such as riparian zones [16]. Some studies have also analyzed variation in body size, wing morphology, and dispersal ability as indicators of ecological adaptations [17], while others have examined variation in Mean Individual Biomass (MIB) in relation to environmental conditions and habitat type [18].

Body size represents one of the key functional traits in the ecology of ground beetles, as it is closely linked to fundamental life-history characteristics and metabolic rate [19,20]. In Carabidae, body size is further associated with wing morphology and the potential for dispersal, reflecting important ecological adaptations to habitat structure and landscape configuration [17]. Such morphological traits provide a basis for developing ecological indicators that integrate information on assemblage structure and enable the assessment of directional changes related to succession and habitat transformation. One such indicator is the Mean Individual Biomass (MIB), defined as the average body mass of individuals within a Carabidae assemblage and widely used as a synthetic measure of functional structure and ecosystem developmental stage [21].

Despite its broad application, relatively few studies have analyzed the significance of MIB in the context of global environmental change. However, shifts in the body size distribution of Carabidae may provide early-warning signals of climate change, habitat fragmentation, or biodiversity loss [22,23,24]. The importance of soil processes and carbon sequestration in assessing ecosystem functioning is increasingly emphasized, which opens new possibilities for interpreting the MIB indicator [25,26].

The aim of this article is to provide a synthetic and critical review of the development of the MIB concept, discuss its applications and methodological limitations, and highlight new perspectives related to global environmental change and ecosystem functioning.

## 2. Methodology of Literature Collection

To collect and review publications related to the Mean Individual Biomass (MIB) index, a comprehensive literature search was conducted between January and March 2024 using the databases Web of Science, Scopus, and Google Scholar.

The following key words and their combinations were used: “Mean Individual Biomass”, “MIB index”, “Carabidae”, “ground beetles”, “succession”, “bioindication”, and “biomass-length relationship”. Only peer-reviewed papers, book chapters, and conference proceedings written in English were included. However, to ensure completeness, relevant sources from the so-called grey literature—such as research reports, dissertations, and publications in national or regional journals not indexed in the Web of Science—were also considered when accessible. Each publication was screened to determine whether the MIB index was explicitly applied as an ecological indicator or discussed in the context of succession, habitat evaluation, or bioindication. The final dataset included 85 publications, some of which directly addressed the application of the MIB index, while others provided theoretical or ecological context related to ground beetle ecology, succession, bioindication, and body length–biomass relationships.

## 3. Development of the Mean Individual Biomass (MIB) Concept

The importance of body size and dispersal ability in ground beetles (Carabidae) for their population dynamics and life-history strategies was anchored in the classical theoretical concepts proposed by den Boer [27] and subsequently developed in his empirical studies on dispersal in Carabidae [28]. This approach provided a solid theoretical foundation for the development of later synthetic indicators, such as the Mean Individual Biomass.

The index was proposed in the early 1980s by Szyszko [21] within the so-called Polish school of carabid ecology, which substantially contributed to the development of functional approaches in bioindication. The concept of MIB emerged from studies on succession in post-agricultural areas and young pine stands, where traditional measures of species diversity failed to reveal clear and consistent developmental patterns. Szyszko suggested that the mean body mass of individuals within Carabidae assemblages could serve as a synthetic indicator of successional stage and ecosystem maturity, representing a methodological shift from taxonomic toward functional traits in ecological assessment [22].

The theoretical background of MIB reflects the observation that early-successional ecosystems are dominated by small-bodied, r-strategist species, whereas late-successional communities are characterized by larger, long-lived K-strategists [27,28]. Integrating these patterns into a single quantitative measure allowed the development of an indicator that is both simple to interpret and tightly linked to successional and functional processes in ecosystems. This functional focus distinguishes MIB from traditional diversity indices and underlies its growing relevance in ecological assessment.

Later works by den Boer [29,30] and den Boer & den Boer-Daanje [31] further expanded and refined concepts, emphasizing the crucial role of dispersal capacity, population stability, and reproductive strategies in shaping the dynamics and structure of Carabidae assemblages. These authors also demonstrated that the classical dichotomy between “spring” and “autumn” breeders is overly simplistic, showing instead that reproductive strategies in ground beetles form a continuum that includes winter-breeding species, species with two reproductive periods per year, and those reproducing throughout much of the annual cycle. The findings from these studies provide an important theoretical framework for the later interpretation of MIB, as they link variation in mean individual biomass to life-history strategies and successional processes.

In practice, MIB is calculated from the biomass of individuals, which in turn is estimated from their body length using regression equations of body length versus mass. The classical equation developed by Szyszko [21] is expressed as:

Ln y = −8.92804283 + 2.5554921 × ln x

where y is the individual biomass (g fresh weight) and x is body length (mm).

This equation was derived empirically by weighing individuals representing a wide range of Carabidae species and has become the reference model for most subsequent studies.

The mean individual biomass of a given assemblage is then calculated according to the formula:

MIB = ∑ y_i_/N

where ∑y_i_ represents the total biomass of all captured individuals and N is their number. Each y_i_ corresponds to the fresh mass of a single beetle, which can be either directly measured or estimated from body length using published regression equations. The MIB value is usually expressed in milligrams of fresh mass per individual and serves as a synthetic measure of the “maturity” of the assemblage.

Although mean body length and Mean Individual Biomass (MIB) are closely related, they describe different aspects of ground beetle (Carabidae) assemblages. As shown in the equation above, the relationship between body length and body mass is non-linear. After transformation, the equation takes the following form:

y = e ^−8.928^ × x ^2.555^

where y is the individual fresh body mass (g), x is the body length (mm), e is the base of the natural logarithm (≈2.71828), used when converting logarithmic equations.

According to this equation, body mass increases faster than body length. For instance, a beetle twice as long may be five or more times heavier. This non-linear relationship makes MIB much more sensitive to the presence of large species than mean body length alone.

**Figure 1:**
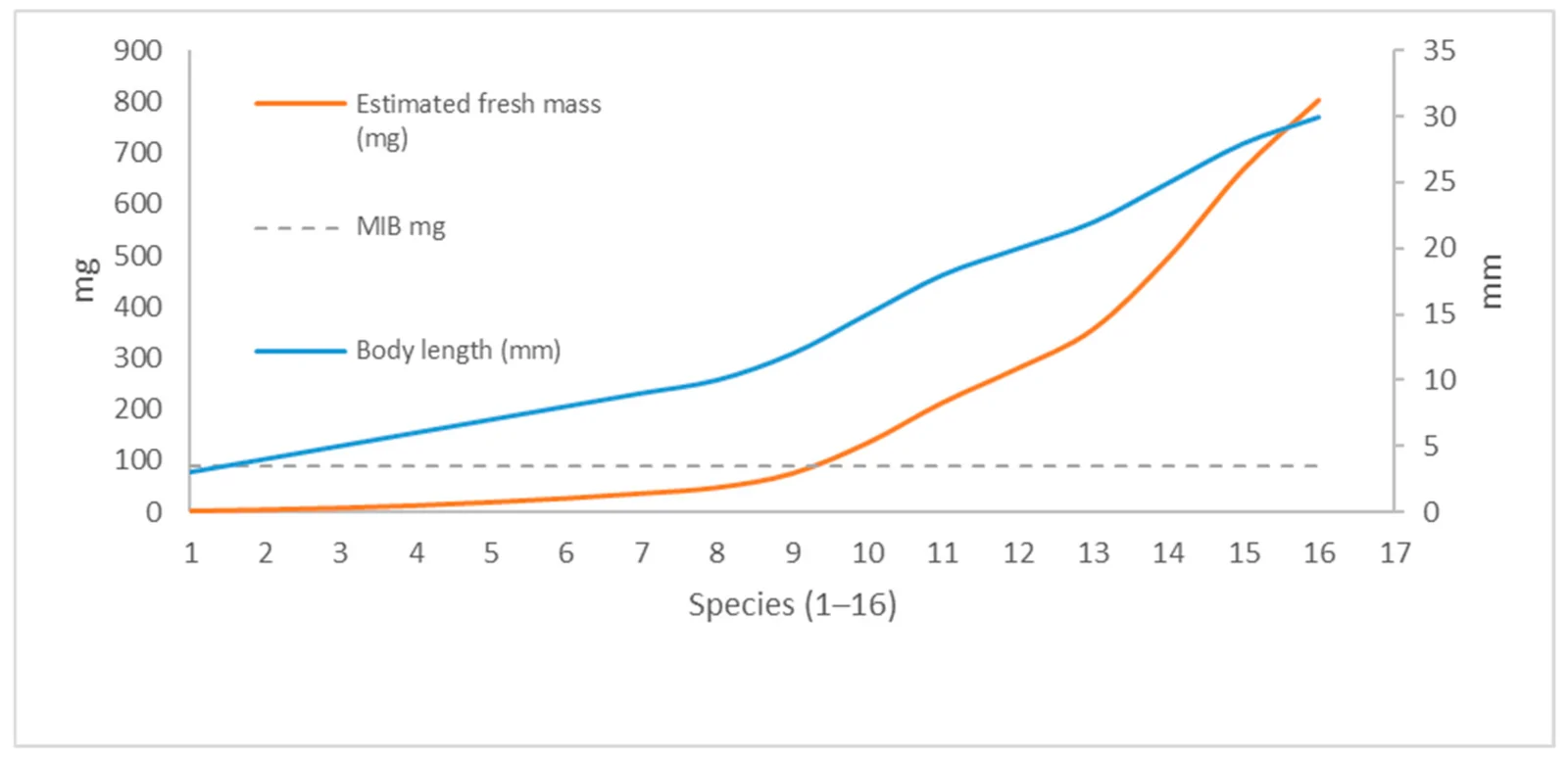
Relationship between body length (mm, right axis) and estimated fresh body mass (mg, left axis) for 16 ground beetle species in a hypothetical assemblage of 50 individuals. The dashed line indicates the Mean Individual Biomass (MIB = 88.8 mg). The use of dual axes illustrates the strongly non-linear increase of biomass with body length while preserving the readability of both variables.

To illustrate this relationship, Figure 1 presents a hypothetical example of MIB calculation for an assemblage of 50 individuals representing 16 ground beetle species of various body sizes. For each species, the mean body length was converted into estimated fresh body mass using the regression equation. The estimated individual masses and MIB in this example are expressed in milligrams (1 g = 1000 mg). In this example, body lengths ranged from 3 to 30 mm, corresponding to estimated fresh masses between 2 and 800 mg. The calculated MIB value for the entire assemblage was 88.8 mg, demonstrating how a small number of large-bodied species can substantially increase the overall MIB despite their low numerical share in the community. The figure clearly demonstrates the non-linear relationship between body length and biomass: as body size increases, biomass rises exponentially. Consequently, the presence or absence of just a few large, late-successional species has a disproportionately strong effect on the resulting MIB value, emphasizing its sensitivity as an ecological indicator.

From an ecological perspective, the Mean Individual Biomass (MIB) integrates both morphological and functional information. Large, long-lived, and often predatory species, usually specialized and with limited dispersal ability, dominate in stable, late-successional habitats such as mature forests. In contrast, small, generalist species with good dispersal ability increase in abundance in early-successional or disturbed habitats [3], resulting in low MIB values. Consequently, MIB reflects not only the average size of individuals but also the size-structured composition of the assemblage and its successional stage [21].

In subsequent years, alternative sets of length–mass equations were developed for different groups of Carabidae and body size ranges [32]. These models, based on empirical measurements of several ground beetle species from the Netherlands, demonstrated that regression parameters can vary considerably among taxa. The resulting diversity of length–mass relationships highlights the need for careful model selection and transparent reporting of the applied equations to ensure comparability among studies [33].

Recent advances, such as the R-function compilation provided by Weiss [34], enable more precise fitting of regression models to specific Carabidae assemblages.

**Figure 2:**
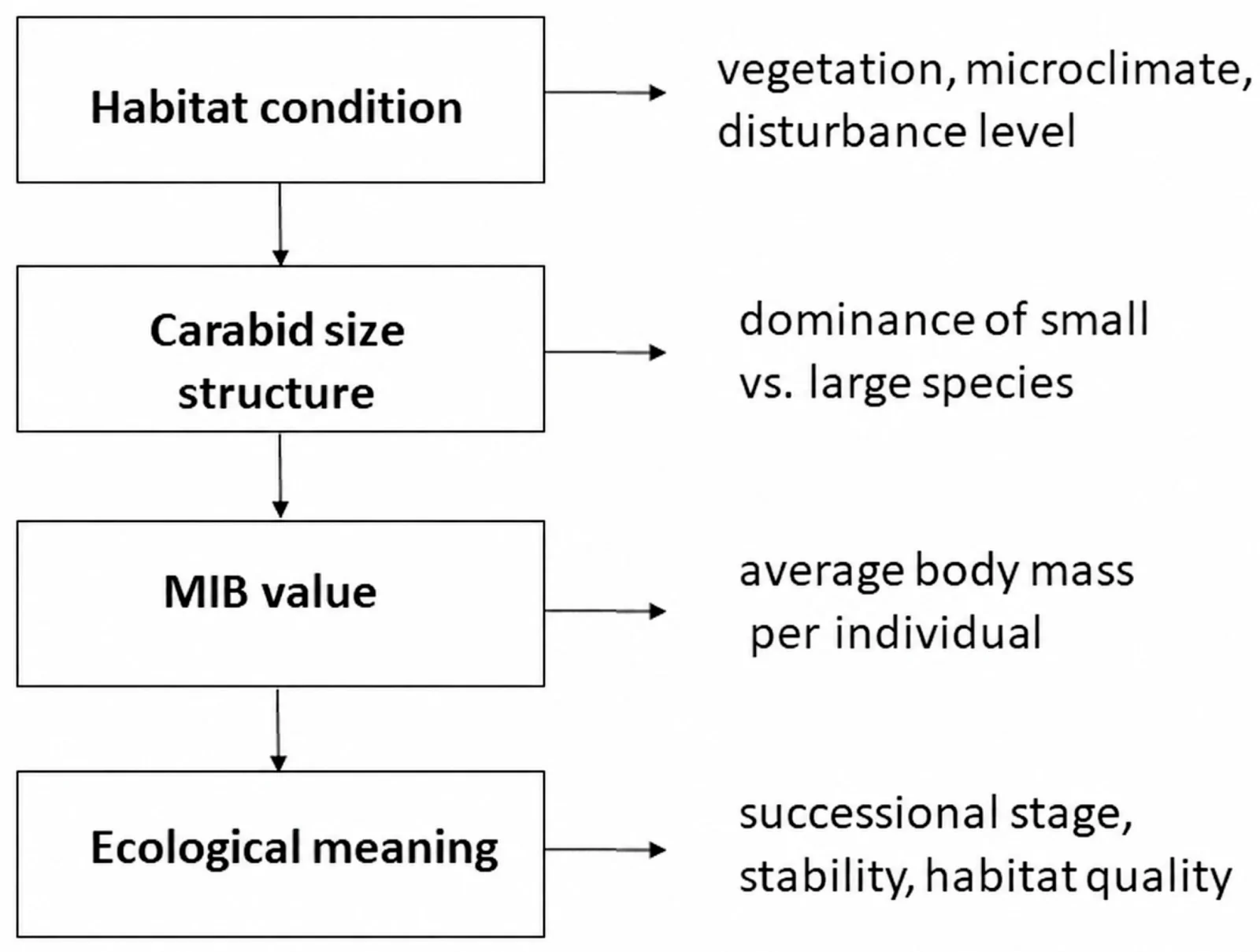
Conceptual framework illustrating the ecological logic of MIB index.

Due to its computational simplicity and intuitive interpretation, the MIB index quickly found application in studies of forest succession, the reclamation of degraded habitats, and the evaluation of the ecological value of forest stands [9,35,36]. Its development represents a successful transition from a functional concept to a widely applied tool in bioindication and applied ecology. The conceptual relationships underlying the MIB index are summarized in Figure 2, illustrating how habitat conditions influence the size structure of ground beetle communities and, consequently, MIB values and their ecological interpretation.

Habitat conditions influence the size structure of ground beetle communities, which determines the MIB value and allows interpretation of successional stage, habitat quality, and ecosystem stability.

## 4. Application of the MIB Index in Different Habitat Types

### 4.1. Forest Succession and Forest Management

The Mean Individual Biomass (MIB) has found its widest application in studies on forest succession and management, particularly in Central Europe. The classical research by Szyszko [21] in pine stands on post-agricultural lands in Poland demonstrated that Carabidae assemblages reflect successional trajectories: early successional stages were dominated by small pioneer species with r-strategy traits, whereas older stands were inhabited by larger, K-strategist species.

This concept was further developed by Schwerk and Szyszko [9], who observed a systematic increase in MIB with stand age and interpreted it as an indicator of secondary succession and increasing ecosystem stability. In later work, Schwerk [37] showed that MIB can also serve as a complementary metric for analyzing changes in carabid assemblages in post-agricultural habitats, although the observed responses were relatively weak and not directly related to successional patterns.

In deciduous forests of Croatia, Jelaska et al. [38] found that MIB values increased with the age of beech stands, and larger species such as *Carabus coriaceus* appeared in mature forests. These results indicate that MIB can also differentiate successional stages in forests with a long developmental history.

Similar patterns have been documented in spruce forests of northeastern Poland, where Nietupski et al. [36] recorded a gradual increase in MIB along a stand-age gradient. Younger stands were characterized by a dominance of small opportunistic species, while older stands contained a higher proportion of large-bodied species typical of stable boreal ecosystems.

Increasing attention has also been given to the relationships between MIB and other components of forest functioning. Kacprzyk et al. [39] found no relationship between MIB and deadwood volume, but reported positive correlations between MIB and soil properties, including soil carbon content, pH, and dehydrogenase activity, suggesting that the index may indirectly reflect processes linked to soil quality and ecosystem functioning.

However, current applications of MIB have focused almost exclusively on forests in temperate and boreal zones. Studies from tropical regions and the Southern Hemisphere are still lacking, which limits the ability to assess the global universality of this indicator and to perform intercontinental comparisons.

### 4.2. Agricultural Landscapes and Field Margins

Beyond forest ecosystems, where the Mean Individual Biomass (MIB) was originally developed and tested, the index has also been applied in agricultural landscapes. These environments, strongly shaped by human activity, provide an important context for assessing the resilience and stability of biotic communities. Existing studies indicate that the Mean Individual Biomass (MIB) can serve as a useful tool for evaluating the effects of land-use intensity and the presence of semi-natural elements in agricultural landscapes. Błaszkiewicz and Schwerk [40] showed that intensively managed arable fields were characterized by the lowest MIB values, whereas higher values occurred in surrounding semi-natural habitats, including field margins and post-agricultural areas. The presence of landscape refuges favored larger, late-successional species, resulting in clearly elevated MIB values compared with agrocenoses dominated by small, highly mobile taxa.

Subsequent studies demonstrated that on post-agricultural lands subjected to natural succession and various reclamation treatments, MIB values increased systematically with the age of sites and the advancement of successional processes [9,37]. The reduction in disturbances and the restoration of semi-natural elements promoted the re-establishment of more stable Carabidae assemblages, reflected in increasing MIB values.

Comparatively, lower MIB values are generally associated with intensively managed croplands, whereas higher values occur in field margins, ecotones, and fallows. These elements act as refuges, enhancing the ecological integrity of agroecosystems.

It should be emphasized, however, that many classical studies on Carabidae in agroecosystems [6,7,41,42,43] focused on morphological and functional traits without directly applying the MIB index. The limited number of studies employing this metric highlights the need for further empirical research across different crop systems and geographic regions.

### 4.3. Post-Industrial and Disturbed Habitats

Post-industrial and degraded areas represent another group of environments where the applicability of the Mean Individual Biomass (MIB) has been tested. These habitats are characterized by strong abiotic disturbances and dynamic secondary succession, making them model systems for assessing ecosystem regeneration.

Studies by Schwerk and Szyszko [9] demonstrated that ground beetles effectively differentiate the degree of habitat transformation in post-industrial areas, with MIB values increasing alongside successional progress, reflecting the appearance of larger, late-successional species. Similar patterns were described by Kędzior et al. [44], who confirmed that rising MIB values accompany the regeneration of post-industrial habitats.

Cárdenas and Hidalgo [45] applied the MIB index to evaluate the regeneration of the Guadiamar ecological corridor in southern Spain, which had been degraded following a mining accident. Their study revealed the sensitivity of MIB to reclamation rate and community stabilization.

Although most studies on disturbed habitats have focused on species diversity (e.g., [46,47]), the available evidence demonstrates that MIB effectively reflects successional trajectories and the recovery of ecological functions. Despite the limited number of studies, this index appears particularly promising for long-term monitoring of reclamation and secondary succession in anthropogenic landscapes.

### 4.4. Glacier Forelands and Extreme Environments

Glacier forelands and other extreme habitats provide unique opportunities for studying primary succession. Harsh abiotic conditions—low soil fertility, unstable substrate, and a short growing season—strongly constrain colonization. In such environments, ground beetles are often among the first colonizers, making them a suitable model group for analyzing the pace of community formation and ecosystem stabilization.

The application of the Mean Individual Biomass (MIB) in glacier forelands has confirmed its usefulness as a successional indicator. Gobbi [48] observed a systematic increase in MIB values with glacier retreat, reflecting the replacement of small pioneering species by larger, late-successional taxa. Comparable patterns were described by Pizzolotto et al. [24] in the Dolomites, where long-term monitoring revealed a shift in Carabidae assemblages associated with the replacement of small, cold-adapted species by larger, thermophilous taxa, both above and below the treeline. Although MIB was not explicitly calculated in that study, the observed community changes are consistent with an increase in mean individual biomass linked to climate warming.

Although several analyses in glacial environments have focused primarily on general patterns of invertebrate succession and community structuring [49,50], the MIB index allows for a synthetic and quantitative assessment of successional dynamics. Available evidence supports its value for evaluating primary colonization and climate-related processes, although current studies remain largely restricted to Europe and should be extended to other regions.

### 4.5. Synthesis of MIB Applications Across Habitats

The reviewed studies demonstrate that MIB has been successfully applied in a wide range of environments—from forests and agricultural landscapes to post-industrial and glacial areas. The index consistently responds to both successional changes and anthropogenic pressure, suggesting a general pattern that may apply across habitats, although most analyses have been conducted in Europe.

To provide a synthetic overview, Table 1 summarizes the general trends in MIB variation across different habitat types and their ecological interpretations. This comparison shows that, regardless of habitat context, MIB serves as a sensitive indicator of successional trajectories and ecosystem stabilization.

## 5. Methodological Issues and Limitations

Although the Mean Individual Biomass (MIB) is a valuable and frequently applied ecological indicator, its application involves several methodological limitations that should be considered both in data interpretation and study design.

### 5.1. Length–Mass Relationships

The calculation of MIB is based on regression models that convert body length of ground beetles into estimated body mass. Weiss and Linde [33] emphasized that the choice of the regression equation can significantly affect biomass estimates, and thus the resulting MIB values. Therefore, reporting the exact equation and parameter values used in MIB calculations is crucial for reproducibility and cross-study comparisons. Recent tools, such as the R-function collection developed by Weiss [34], facilitate the selection of appropriate models and enhance the comparability of studies.

### 5.2. Seasonal and Phenological Variability

Carabidae assemblages exhibit pronounced annual variability: small species tend to dominate in spring, whereas larger taxa are more frequent in autumn. As noted by Thiele [51] and Lövei and Sunderland [52], phenology and reproductive strategies can strongly influence community structure. Short-term sampling may therefore yield incomplete results, and reliable MIB analyses require long-term datasets covering the full activity season. It should also be noted that most Carabidae species overwinter as adults, while others overwinter as larvae at different developmental stages. The overwintering instar determines the timing of adult emergence in the following season, which should be considered when selecting optimal sampling periods.

### 5.3. Sampling Methods

The most commonly used Barber pitfall traps measure activity density rather than absolute population abundance [53,54]. Consequently, MIB values can also be shaped by the sampling method itself. Studies have shown that both trap design [55] and the type of preservative liquid used [56] may influence the composition of captured Carabidae. Comparative assessments of different trapping techniques [57] have nevertheless confirmed that Barber traps remain the most effective method for monitoring, provided that protocols are properly standardized.

### 5.4. Minimum Sample Size

The reliability of MIB values depends on the number of individuals collected. Schwerk and Szyszko [58] indicated that when fewer than approximately 50 individuals are captured, MIB estimates become highly uncertain. In such cases, the presence of a single large or very small species can substantially bias the mean, complicating the interpretation of results.

### 5.5. Sensitivity to Community Structure

MIB synthesizes information about the community into a single value, which may obscure details related to particular species or overall functional diversity [3,4]. Moreover, a mass occurrence of a single large-bodied species (e.g., *Carabus* spp.) can substantially increase MIB values, not necessarily reflecting the actual successional stage [58].

### 5.6. Spatial Scale and Habitat Heterogeneity

As an averaged metric, MIB may mask small-scale variation in mosaic landscapes. Local values can differ substantially depending on microhabitat conditions. Magura et al. [59] analyzed changes in the proportion of small and large ground beetle species along an urbanization gradient. Although their study did not directly refer to MIB, the observed shift toward smaller-bodied, generalist species in more urbanized areas supports interpretations based on body-size distribution patterns.

### 5.7. Summary

In summary, the application of MIB requires careful methodological standardization and transparent data reporting. To facilitate comparability and reduce interpretation bias, Table 2 summarizes a proposed minimal standard for reporting and quality control in MIB-based studies.

## 6. MIB in the Context of Global Change and Anthropogenic Pressure

The Mean Individual Biomass (MIB) of ground beetles, originally developed for studies of ecological succession, has increasingly been applied to analyses of global environmental change and anthropogenic impact. By integrating information on species composition and body size, the index captures complex responses of *Carabidae* assemblages to climatic, soil-related, and human-induced factors.

### 6.1. Climate Change and Species Traits

Phenological shifts and glacier retreat have led to systematic changes in Carabidae community structure. In alpine glacier forelands, MIB has been shown to increase predictably along successional gradients as habitats stabilize [48], while long-term trends indicate a shift toward smaller, more mobile species [24]. More recent studies suggest that extreme weather events, such as prolonged droughts, can cause sharp declines in abundance and biomass, particularly among large, less mobile species [60,61,62]. These findings suggest that MIB has the potential to indicate early functional shifts in beetle communities under climate change, provided that long-term datasets cover entire activity seasons and consistent sampling protocols are applied across years.

### 6.2. Soil Processes and Carbon Sequestration

As terrestrial ecosystems undergo succession, the accumulation of organic matter and the improvement of soil structure enhance the capacity for soil organic carbon (SOC) storage. In mountain forests, MIB values have been observed to increase along successional gradients and to co-occur with higher soil pH, dead wood resources, and SOC content but not with deadwood volume [39]. Similar relationships were reported by Szyszko-Podgórska et al. [63,64], who highlighted links between soil macrofauna activity, litter decomposition, soil organic carbon, and, in the case of Carabidae and butterflies, assemblage structure in relation to soil characteristics and plant diversity. On this basis, Szyszko et al. [65] proposed the use of animals, including the Mean Individual Biomass of Carabidae and selected bird species, as indicators of landscapes valuable for biodiversity and ecosystem services, particularly carbon sequestration, rather than demonstrating a direct statistical relationship between MIB and carbon stocks. Although these correlations require further validation, they show that MIB tends to be higher in habitats with greater soil organic carbon content, most likely due to shared environmental drivers such as vegetation structure, microclimate, and litter dynamics. Thus, MIB should be regarded as an ecological correlate rather than a direct proxy for carbon sequestration. Global syntheses on SOC storage and soil functions [66,67] and conceptual work on the role of soil fauna in organic matter dynamics [25] have emphasized the importance of soil organic matter stabilization and fauna-driven processes in carbon cycling, thereby strengthening the ecological context for MIB applications.

### 6.3. Anthropogenic Pressure and Landscape Transformation

Intensive agriculture, urbanization, and habitat fragmentation typically lead to declines in MIB values through the elimination of large, late-successional species and the dominance of small-bodied generalists. In Europe, urbanization has promoted community homogenization and the loss of large species [2,68,69]. Similar trends have been observed in highly transformed landscapes [23,70]. More recent studies [71,72,73] confirm these patterns, documenting reduced diversity, shifts toward small eurytopic species, and localized increases in Carabidae biomass in landscapes with a higher proportion of woody elements.

### 6.4. Synthesis

Current evidence indicates that MIB can effectively reflect the responses of Carabidae assemblages to climatic, soil, and anthropogenic drivers. Although the index does not replace direct measurements of soil processes, it provides valuable indirect information on habitat quality and may serve as an early signal of changes in ecological stability under global environmental change.

## 7. Integration of MIB with Other Indicators

In recent years, increasing attention has been devoted to integrating the MIB with other approaches for assessing biodiversity and ecosystem functioning. In the context of global environmental change, such integrated frameworks provide a more complete, mechanistic understanding of ecological processes.

### 7.1. Classical Diversity Indices, Traditional Taxonomic Diversity Metrics

Traditional taxonomic diversity metrics, such as the Shannon, Simpson, and Jaccard indices, effectively reflect species richness and community evenness; however, they do not account for functional aspects of assemblages. These indices have been widely applied in analyses of Carabidae communities across various habitat types. Riley and Browne [74] demonstrated that they can distinguish ground beetle assemblages according to forest age. Allegro and Sciaky [75] found that, despite accounting for both species richness and evenness, the Shannon and evenness indices did not effectively capture successional or structural differences in poplar stands, which led the authors to propose a new, more ecologically oriented measure—the Forest Affinity Index (FAI).The FAI quantifies the similarity of a given carabid assemblage to reference forest communities by weighting the relative frequency of each species according to its forest specialization value and the total number of species in the sample. Similarly, Porhajašová et al. [76] and Kowalska et al. [77] applied Shannon-based indices to assess Carabidae diversity in agricultural systems, while Bouraga et al. [78] used them in desert ecosystems, highlighting their sensitivity to environmental and climatic factors. In turn, Szyszko-Podgórska et al. [79] applied the Simpson and Jaccard indices to analyze the diversity of Carabidae in agricultural and urbanized landscapes, identifying key soil variables that shape community structure. Classical diversity indices therefore primarily capture taxonomic composition and evenness, but do not account for functional traits such as life-history strategy, trophic position, body size, or dispersal ability. Indicators based on these traits, including the Mean Individual Biomass (MIB), provide a more comprehensive understanding of ecological processes within Carabidae assemblages.

### 7.2. Functional Traits and Ecological Strategies

In recent years, there has been a growing interest in indicators based on functional traits, such as dispersal ability, wing morphology, and body size. Philpott et al. [80] demonstrated that both local and landscape-scale factors influence the body size and dispersal potential of Carabidae in urban gardens. Maveety and Browne [81] documented variation in body length and wing form along altitudinal gradients in the Andes. Do and Choi [71] confirmed that urbanization shapes both the flight ability and trophic types of ground beetles, while a global synthesis by Hahs et al. [82] revealed that cities favor small, mobile, generalist species. Additionally, Charalabidis et al. [83] indicated that accounting for trophic differentiation—for instance, between granivorous and omnivorous species—can substantially enhance the functional interpretation of ecosystem studies. Similarly, Gobbi and Fontaneto [84] highlighted that species richness alone may be a misleading parameter when assessing human impacts on ground beetle assemblages, recommending the use of morpho-ecological traits such as wing morphology, diet, and body size as more reliable ecological indicators.

In this context, the Mean Individual Biomass (MIB) represents a synthetic, morphology-based indicator that integrates information on body size and community structure, enabling a quantitative assessment of successional changes and environmental pressures.

### 7.3. Molecular Methods

The rapid development of molecular techniques, including DNA metabarcoding and environmental DNA (eDNA) analysis, has opened new possibilities for rapid biodiversity monitoring [85,86]. Although these methods provide precise information on species composition, they do not allow for direct assessment of the functional aspects of communities, such as biomass, body size, or trophic structure. In this context, the Mean Individual Biomass (MIB) represents a complementary approach, adding a functional and ecological dimension that is often missing from molecular analyses.

The most recent approach, known as “Fun-eDNA” [87], combines environmental DNA data with information on species functional traits, enabling large-scale assessments of functional diversity across multiple trophic groups. This method highlights the strong potential for integrating MIB-based analyses with molecular indicators, allowing for a more comprehensive understanding of ecosystem functioning under global environmental change.

### 7.4. The Index of Natural Value (INV)

In addition to the Mean Individual Biomass (MIB), several other synthetic indicators have been developed to assess habitat quality and ecological integrity. One of the examples is the Index of Natural Value (INV), first introduced by Pizzolotto [88] and later refined by Brandmayr et al. [89]. This index combines species richness with important functional traits, such as endemism, brachyptery, and trophic specialization, providing a measure of habitat naturalness and conservation importance.

Recent work by Peretti et al. [90] showed that the INV can be effectively applied to map biodiversity and evaluate habitat transformation in Alpine environments. Because it considers both taxonomic and functional aspects of communities, the INV complements MIB by offering a broader picture of habitat integrity and ecological value.

However, as noted by Pizzolotto [88] and Brandmayr et al. [89], the INV depends on expert-based evaluation of species traits and requires regional calibration, which may limit its direct comparability across studies or biogeographical regions. Nevertheless, when considered together, MIB and INV may provide a promising framework for bioindication, potentially integrating biomass-based and trait-based perspectives on ecosystem functioning under environmental change.

### 7.5. The Importance of an Integrated Approach

Kotze et al. [2] emphasized that a comprehensive understanding of Carabidae dynamics requires combining multiple indicators—from classical diversity measures to functional traits and soil parameters. Recent studies on the role of soil fauna in carbon sequestration [25] suggest that integrating MIB with soil and vegetation analyses can substantially enhance its value in research on ecosystem services and climate change.

Current evidence thus indicates that the greatest potential of MIB lies not in its isolated use but in its integration with other indicators, allowing for simultaneous assessment of taxonomic diversity, functional processes, and environmental context. Integrating MIB with complementary bioindication approaches enables a more holistic understanding of complex ecological processes by combining data on species composition, functional traits, and community genetic structure. This integrated framework offers a promising direction for future biodiversity monitoring and for linking MIB-based analyses with broader ecosystem functions.

To summarize these relationships, Table 3 presents the main advantages, limitations, data requirements, and common interpretative pitfalls of the most frequently used biodiversity indicators and assessment approaches.

## 8. Future Perspectives and Research Agenda

Despite four decades of research on the Mean Individual Biomass (MIB), its potential has not yet been fully realized. To date, most analyses have focused on forests, agroecosystems, post-industrial areas, and glacier forelands, where its usefulness in tracking successional changes and assessing habitat stability has been confirmed [21,24,44]. Studies conducted in post-industrial areas have shown that MIB effectively differentiates regeneration stages and can serve as an indicator of ongoing ecological processes [44]. An important future direction involves examining the effects of urbanization and habitat fragmentation. It has been shown that urbanized areas tend to select for small, mobile, generalist species, which is reflected in reduced MIB values [71,82]. These findings highlight the need for further research in highly transformed landscapes, where the significance of the index remains insufficiently recognized.

Another area requiring deeper investigation is the integration of MIB with soil and climatic data. Positive correlations have been reported between MIB values, deadwood abundance, and soil organic carbon (SOC) content in Central European forests [39]. Although these results confirm the potential of MIB as an indirect indicator of soil processes, the issue remains largely open and calls for comparative studies across other regions and ecosystem types. Broader syntheses [25,26,67] emphasize the role of soil fauna and environmental factors in carbon sequestration, providing a rationale for future efforts to integrate MIB with soil and vegetation analyses.

To enhance the practical relevance of MIB, it should be incorporated into existing biodiversity and climate monitoring schemes. When combined with classical diversity indices (Shannon, Simpson), functional indicators (dispersal ability, wing morphology, trophic strategies), and molecular methods [85,86], MIB can serve as an integrative tool. Table 4 summarizes the strengths, limitations, and integration potential of various biodiversity assessment approaches, highlighting that the greatest value of MIB lies not in its isolated application but in its integration with complementary methods.

Based on the above considerations, several key directions for future research can be identified:

(1) verification of the applicability of MIB in tropical and non-European landscapes, where comparative data are still lacking;
(2) analysis of relationships between MIB values and soil processes, particularly carbon sequestration, through experimental and long-term studies;
(3) development and standardization of methodologies, including body length–mass equations and the use of new databases and bioinformatic tools;
(4) integration of MIB with molecular analyses and functional indicators to establish more comprehensive frameworks for biodiversity and habitat quality monitoring.

## 9. Conclusions

The Mean Individual Biomass (MIB) has been a breakthrough indicator in Carabidae ecology, combining computational simplicity with intuitive interpretation. Available studies show that MIB effectively reflects successional changes in forests, agroecosystems, and post-industrial landscapes, while also responding to pressures of urbanization and habitat fragmentation. Increasing evidence also indicates its association with soil processes, including carbon sequestration, highlighting the broader bioindicative potential of this metric.

Methodological limitations—such as the lack of full standardization of length–mass equations, the influence of pitfall trap design, seasonal variability, and the need for a minimum number of specimens—demonstrate that the application of MIB requires caution and harmonization of research approaches. Only standardized protocols will enable its broader use in comparative analyses and international meta-studies.

The greatest developmental potential of the index lies in its integration with other approaches—classical diversity indices, functional trait analyses, molecular techniques, and soil and climate studies. Expanding research beyond Europe will allow verification of its universality and relevance across diverse biogeographical contexts.

In summary, MIB may evolve from a specialized tool for studying ecological succession into a robust, yet context-dependent indicator of habitat quality and ecosystem functioning in the era of global change.

## Figures and Tables

**Table 1:** Trends in MIB values across habitat types, regions, and their ecological interpretation.

Habitat Type	Region/Scale	Trend in MIB Values	Ecological Interpretation/Notes
Forests—secondary succession	Central and Southern Europe	↑ with stand age	Reflects transition from small pioneer species to large, late-successional taxa; no data available from tropical regions
Agricultural landscapes/field margins	Central Europe	Fields: ↓; refuges: ↑	Sensitive to land-use intensity and presence of semi-natural refuges; relatively few studies available
Post-industrial habitats	Poland, Spain	↑ during regeneration	Accurately reflects habitat recovery and reclamation processes; number of studies still limited
Glacier forelands and extreme environments	European Alps	↑ with stabilization	Indicator of primary succession and climate-related changes; most data originate from Alpine and other European glacial forelands
Urbanization/fragmentation	Europe, global	Generally: ↓	Selection for small, mobile generalist species and assemblage homogenization; requires studies outside Europe

Note: Arrows indicate the general trend in MIB values (↑ increase, ↓ decrease).

**Table 2:** Recommended minimum reporting standards and quality control in MIB-based studies.

Element/Issue	Why it Matters	What to Report/Minimum Standard
Length–mass equations	The choice of regression model affects biomass estimation and comparability among studies.	Provide the exact equation(s) used, length range, and source; if mixed models are applied, specify assignment rules; indicate the software or tool used (e.g., R script).
Barber traps—design and preservative liquid	Pitfall traps measure activity density rather than absolute abundance; trap design and preservative type may selectively influence species composition.	Report trap diameter and depth, cover type, preservative used, exposure duration, and inspection frequency; note any corrections applied.
Sampling effort and season	Species phenology causes seasonal variation in MIB values.	Full-season or multi-year sampling recommended; report sampling schedule and total effort (trap-days).
Minimum sample size	Small samples produce unstable and difficult-to-interpret MIB values.	Report number of individuals (N) and species; ≥50 individuals per comparison unit recommended, or aggregate data across time/space.
Spatial/temporal unit	Averaging may obscure habitat mosaic patterns and local differences.	Provide exact plot size, trap spacing and number, number of replicates, and their spatial arrangement.
MIB calculation method	Reproducibility requires clear description of the computation process.	Specify whether biomass was measured directly or estimated via length–mass regression; indicate aggregation method (weighted or unweighted mean) and units used.
Habitat context	Interpreting MIB without environmental data may be misleading.	Include vegetation structure, dead wood volume, and soil parameters (e.g., SOC, pH), when available.
Interpretation caution	Dominance of large-bodied species (e.g., *Carabus* spp.) may artificially increase MIB.	Report dominant species and their relative abundance; consider complementary indicators (e.g., functional traits or diversity indices).

Note: The table summarizes key methodological aspects influencing the reliability and comparability of studies based on the MIB index. It emphasizes the importance of transparent reporting and standardized sampling procedures to ensure data reproducibility across different habitats and regions.

**Table 3:** Characteristics of biodiversity indicators and approaches in relation to MIB.

Indicator/Approach	Advantages/Strengths	Limitations	Data Requirements and Costs	Common Interpretative Pitfalls
MIB (Mean Individual Biomass)	Simple to calculate; synthetically reflects successional changes; intuitive to interpret.	Sensitive to small sample sizes; lack of standardized length–mass equations; method-dependent (pitfall traps).	Body length data and L–M equations; sufficient sample size (N > 50).	Overinterpretation as a universal measure of “ecosystem maturity”; comparing results without specifying the applied L–M equation.
Diversity indices (Shannon, Simpson, Jaccard)	Accurately capture species richness and evenness; widely used and comparable across studies.	Do not account for functional traits; limited interpretation of ecological processes.	Full species-level identification across assemblages.	Reducing ecological complexity to a single number; neglecting functional dominance patterns.
Functional traits (body size, wings, trophic type)	Reflect underlying ecological mechanisms; high interpretative value for ecosystem functioning.	Time-consuming trait assessment; challenges in standardizing trait categories.	Morphological and ecological trait data for multiple species.	Ignoring intraspecific variability; overgeneralization of trait categories.
Trophic strategies	Explain the role of Carabidae in nutrient cycling and biological control.	Require feeding experiments; difficult to upscale across habitats.	Dietary data, feeding trials, or isotopic analyses.	Treating species as trophically uniform without accounting for variability.
Molecular methods (DNA metabarcoding, eDNA)	Rapid and precise species identification; allows environmental sample analysis.	Lack functional information; dependent on the quality of reference databases.	Laboratory analyses, sequencing, and bioinformatics expertise.	Confusing DNA presence with actual occurrence or abundance of individuals in the assemblage.
INV (Index of Natural Value)	Integrates taxonomic richness with functional traits (e.g., endemism, brachyptery, trophic specialization); enables assessment of habitat naturalness and conservation value.	Requires expert-based trait evaluation; functional weighting partly subjective; still limited applications outside specific regions (mainly Italy).	Comprehensive species lists with ecological trait data; expert ecological scoring.	Assuming cross-regional comparability without calibration; interpreting INV as a direct biodiversity measure instead of naturalness.

Note: The table emphasizes that no single approach can fully capture the complexity of ecological processes. The highest interpretative potential is achieved by combining MIB with taxonomic, functional, and molecular indicators.

**Table 4:** Potential for integrating different biodiversity assessment approaches with MIB.

Indicator/Approach	Integration Potential with MIB
Diversity indices	Combining MIB with diversity metrics allows linking species richness with the dynamics of functional traits.
Functional traits	Integration enables the association of mean body size with other traits such as dispersal ability and wing morphology.
Trophic strategies	MIB can be combined with trophic group analyses to assess the functional stability of communities.
Molecular methods	Provide rapid identification of species composition, while integration with MIB adds a functional interpretation.
Index of Natural Value (INV)	The combined use of MIB and INV may provide a complementary bioindication framework, linking biomass-based and trait-based perspectives on habitat integrity.
Soil and climatic parameters	Integration with environmental data (SOC, moisture, temperature) allows MIB to be used as an indicator of ecosystem services.

## Data Availability

No new data were created or analyzed in this study. Data sharing is not applicable to this article.

## Abbreviations

The following abbreviations are used in this manuscript:

MIB	Mean Individual Biomass
SOC	Soil Organic Carbon
FAI	Forest Affinity Index
FS	Flower Strip
DNA	Deoxyribonucleic Acid
eDNA	Environmental DNA
